# Influenza A Virus Infection Reactivates Human Endogenous Retroviruses Associated with Modulation of Antiviral Immunity

**DOI:** 10.3390/v14071591

**Published:** 2022-07-21

**Authors:** Hengyuan Liu, Valter Bergant, Goar Frishman, Andreas Ruepp, Andreas Pichlmair, Michelle Vincendeau, Dmitrij Frishman

**Affiliations:** 1 Department of Bioinformatics, Technical University of Munich, 85354 Freising, Germany; hengyuan.liu@tum.de; 2 Institute of Virology, School of Medicine, Technical University of Munich (TUM), 81675 Munich, Germany; v.bergant@tum.de (V.B.); andreas.pichlmair@tum.de (A.P.); 3 Institute of Experimental Genetics, Helmholtz Zentrum München, German Research Center for Environmental Health (GmbH), 85764 Neuherberg, Germany; goar.frischmann@helmholtz-muenchen.de (G.F.); andreas.ruepp@helmholtz-muenchen.de (A.R.); 4 German Center for Infection Research (DZIF), Munich Partner Site, 81675 Munich, Germany; 5 Research Group Endogenous Retroviruses, Institute of Virology, Helmholtz Zentrum München, 85764 Neuherberg, Germany

**Keywords:** gene regulation, expression analysis, genome analysis, functional annotation, immune response, virus-host interactions

## Abstract

Human endogenous retrovirus (HERVs), normally silenced by methylation or mutations, can be reactivated by multiple environmental factors, including infections with exogenous viruses. In this work, we investigated the transcriptional activity of HERVs in human A549 cells infected by two wild-type (PR8M, SC35M) and one mutated (SC35MΔNS1) strains of Influenza A virus (IAVs). We found that the majority of differentially expressed HERVs (DEHERVS) and genes (DEGs) were up-regulated in the infected cells, with the most significantly enriched biological processes associated with the genes differentially expressed exclusively in SC35MΔNS1 being linked to the immune system. Most DEHERVs in PR8M and SC35M are mammalian apparent LTR retrotransposons, while in SC35MΔNS1, more HERV loci from the HERVW9 group were differentially expressed. Furthermore, up-regulated pairs of HERVs and genes in close chromosomal proximity to each other tended to be associated with immune responses, which implies that specific HERV groups might have the potential to trigger specific gene networks and influence host immunological pathways.

## 1. Introduction

The Influenza A virus (IAV) continues to cause a large number of deaths and hospitalizations worldwide. IAV infections are often accompanied by a strong inflammatory response [1], which is critical for controlling viral replication but also contributes to lung injury, morbidity, and death [1]. Thus, it is important to understand the cellular processes associated with IAV infection and to identify the factors that trigger the cellular immune response.

Recent research indicates that human endogenous retroviruses (HERVs) are involved in the regulation of immune functions [2,3,4,5]. Approximately 8% of the human genome consists of HERV sequences [6], which are integrated into the genome as complete or partially-truncated elements [7]. In addition, up to 500,000 copies of HERV-derived solitary “long terminal repeats” (LTRs) are scattered across the genome [7,8]. HERV LTRs often encode cis-regulatory elements, which can regulate host gene activity via multiple mechanisms and rewire regulatory networks when reactivated [6]. Although most HERV elements are silenced by mutations or epigenetically controlled, they can be reactivated by environmental conditions, including infections with exogenous viruses such as HIV-1 [9], hepatitis C [10,11], or IAV [12,13]. Upon reactivation, HERVs can impact the antiviral immune response via multiple mechanisms. HERV-derived nucleic acids can induce pattern recognition receptors, such as retinoic-acid-induced gene-1-like (RIG) receptors or toll-like receptors (TLR) [14]. Interestingly, one prominent antiviral response against IAV is based on the RIG-I pathway [15]. Moreover, HERV-derived proteins, such as the HERV-W envelope protein, have been reported to induce cytokine production via TLR signaling [16,17]. Several recent reports have shown that HERV-derived proteins can also stimulate the adaptive immune system by inducing a T or B-cell response [18,19]. Moreover, HERV-derived long non-coding RNAs (lncRNA) can have immunostimulatory functions [20], and HERV-derived promoters or enhancers can impact the expression of inflammatory genes, which influence antiviral immunity [2,6,21].

We here investigated the extent to which IAV infection can influence HERV expression and thereby regulate host gene networks and, in turn, affect the antiviral immune response. We analyzed changes in the expression of HERVs as well as cellular genes in the RNAseq datasets from A549 cells infected with three different IAVs: influenza A/PR/8/1934(H1N1) (PR8M), influenza A/seal/Mass/1-SC35M/1980 (H7N7) (SC35M), as well as an NS1 deleted version of the latter strain (SC35MΔNS1). This analysis revealed multiple HERV elements as well as HERV LTRs that are activated upon IAV infection, including LTR5_Hs, LTR5, and LTR12C, already known to impact immunological processes. Moreover, we identified differentially expressed genes within 100kb from a differentially expressed HERV LTR (the so-called DEHERV-G pairs [22]). According to the functional enrichment analysis, a significant proportion of such DEHERV-G pairs involve genes associated with immune response pathways. Thus, our data suggest that immune regulatory circuits are governed by HERVs and regulated upon IAV infection due to changes in HERV expression.

## 2. Materials and Methods

### 2.1. Transcriptomic Analysis of IAV-Infected A549 Cells

Transcriptomics data on IAV-infected cells were obtained from the study of Bergant and Pichlmair et al. (manuscript in preparation). To evaluate IAV-induced transcriptional and post-transcriptional changes, Bergant and Pichlmair performed deep RNA sequencing on A549 cells infected in three separate wells for 24 h with IAV strains A/Puerto Rico/8/1934 H1N1 (PR8), A/seal/Mass/1-SC35M/1980(H7N7) (SC35M) [23], or SC35M with genetic ablation of NS1 (SC35MΔNS1) [24] at MOI 3. Infecting the cells at the same MOI allows the viruses to propagate in their natural way. The virus stocks were produced in Vero E6 cells as previously described [25]. Cellular RNA was isolated using a Macherey-Nagel NucleoSpin RNA mini kit according to manufacturer instructions and submitted for single-end RNA sequencing on the Illumina NextSeq 2000 platform with a read length of 75 bp.

### 2.2. HERV Annotation

From the Human Endogenous Retroviruses Database (HERVd) [26] we obtained HERV annotation, including the ID and coordinates of each HERV locus, as well as the assignments of internal portions and LTRs of HERV elements to one of the five top-level superfamilies (ERV1, ERV2, ERV3, Gypsy, and Unclassified). The HERVd does not provide any information on the association between the internal portions of the proviruses and their LTRs. To establish such an association, we used the information provided by Kojima in Table 4 in [27], in which the five superfamilies are further subdivided into 22 groups. For example, the ERV3 superfamily contains the following groups: HERVL, HERVS, MaLR, and Unclassified. Within each group, a list of internal portions and their associated LTRs is provided. For some HERV element types omitted in the Kojima publication, we conducted additional manual annotation using the DFAM database [28]. We ignored 2 types of internal regions not associated with any LTRs and 122 LTR types not associated with any internal regions. This annotation effort resulted in 449 types of HERV elements containing 103 different types of internal portions and 346 types of LTRs (Table S1), covering a total of 458,075 HERV loci.

### 2.3. RNA-Seq Data Processing

Quality control and preprocessing of the RNA-seq data were performed by fastp [29] with all default parameters. Single-end reads were mapped to the human reference genome (assembly GRCh38) using the RSEM package with the “-star” parameter [30], i.e., using the STAR aligner [31]. RSEM automatically sets proper parameters for STAR so that the multi-mapped reads are not discarded. RSEM is designed specifically for dealing with multi-mapped reads at the gene level or isoform level using the expectation maximization algorithm. We built the count matrices for genes and HERV loci separately across all samples using “expected counts” from the RSEM output, which are the estimated maximum likelihood values of different genes or HERV loci. This could help to avoid massive underestimation of HERV expression compared with the studies that use only uniquely mapping reads for HERV expression analysis. Gene annotation was obtained from the Ensembl database (version 99) [32]. To measure the overall expression of HERV elements for individual samples, the approach described by Bhardwaj et al. was adopted (Bhardwaj et al., 2015). We obtained consensus sequences of HERV elements from the Dfam database [28] and created a faux “HERV genome” containing all of the consensus sequences for these elements. The RNA-Seq data were mapped to the faux genome with RSEM, and the expected read counts were used to build a new count matrix. Subsequently, the read count matrices of genes and consensus sequences of HERV elements were merged together for later analysis, while the HERV loci matrix was analyzed separately. The genes, HERV loci, or consensus HERV elements that did not have reads mapped to them at least in one sample were excluded from consideration.

### 2.4. Differential Expression Analysis

The differential expression (DE) analysis of gene loci, HERV loci, and HERV elements was conducted using the DESeq2 package [33]. The HERV loci count matrix and the merged count matrix containing genes and different types of HERV elements were modeled by a negative binomial distribution, with a design matrix to identify the different conditions (including cell type and infection state) associated with each sample. Log2 fold change (LFC) values produced by DESeq2 were used to compare the expression between the treatment and reference samples. Subsequently, the LFC values were shrunk using the “apeglm” algorithm [34], and the shrunk LFCs > ±1 with s-values of <1 × 10^−3^ [35] were considered indicative of differential expression. While assessing the overall expression of HERV elements, we excluded the elements with the average value of the read counts (further referred to as basemean) across all samples lower than 10.

### 2.5. Association of Up-Regulated HERV-LTR Types with DEGs

Based on the LTR type-specific differential expression analysis described above, we focused on 18 types of HERV LTRs (Table S2), each of which was either up-regulated or belonged to a HERV provirus whose internal portion was up-regulated. For each HERV-LTR type, we performed a Fisher’s exact test to examine whether the individual differentially expressed loci of this particular type are significantly associated with DEGs (Table 1). Since HERV LTRs may harbor enhancers, which take effect regardless of the relative position (upstream or downstream) or direction with respect to the target genes [36], in this test, we did not require HERV LTRs to be located upstream and on the same strand relative to the target genes. The result of the Fisher’s test with a significant (Benjamini–Hochberg corrected *p*-values < 0.05) odds-ratio larger than 1 implies that the differentially expressed loci belonging to the given HERV-LTR type occurred within 100 kbp of DEGs’ TSS more frequently than expected by chance and is interpreted as a positive association between DEGs and DEHERV loci of this type.

### 2.6. Motif Analysis of Up-Regulated HERV-LTRs

Motif analysis was performed using HOMER 4.11 [37]. HOMER screens all possible oligos of a certain length in the “target sequence set” along with the “background sequence set” and determines motif enrichment based on the cumulative hypergeometric distribution. We used the “findMotifsGenome.pl” script from HOMER and provided it with the coordinates of selected up-regulated HERV-LTR loci as a target set. The background sequence set was automatically selected by HOMER. The “-mask” parameter was not used since our targets are repetitive sequences.

### 2.7. Identification of Differentially Expressed HERV and Gene Pairs (DEHERV-G Pairs)

Following [22], for each differentially expressed HERV locus, we identified the nearest DE gene within 100 kbp on the same strand. The distance between a HERV locus and a gene locus was defined as the distance between their closest edges (transcript start site or transcript end site). For HERV loci situated within gene loci, the distance was considered to be 0. The protein-coding genes occurring in the DEHERV-G pairs were subjected to functional enrichment analysis.

### 2.8. Functional Enrichment Analysis

GO (Gene Ontology) and KEGG (Kyoto Encyclopedia of Genes and Genomes) enrichment analysis of the differentially expressed protein coding-genes were performed using the modEnrichr suite [38].

## 3. Results

### 3.1. Identification of Differentially Expressed Genes (DEGs) and HERVs (DEHERVs)

To explore whether IAV infection causes changes in HERV expression as well as the expression of host genes, we infected A549 cells with two different IAV strains, PR8M and SC35M. Moreover, we included the IAV mutant “ΔNS1” (SC35MΔNS1), as the NS1 protein is known to suppress the host’s innate immune response by interfering with the expression of human antiviral factor PAF1, allowing the virus to replicate more efficiently [39,40]. Then, 24 h post-infection, we extracted the total RNA, which was subjected to single-end RNA sequencing.

**Figure 1:**
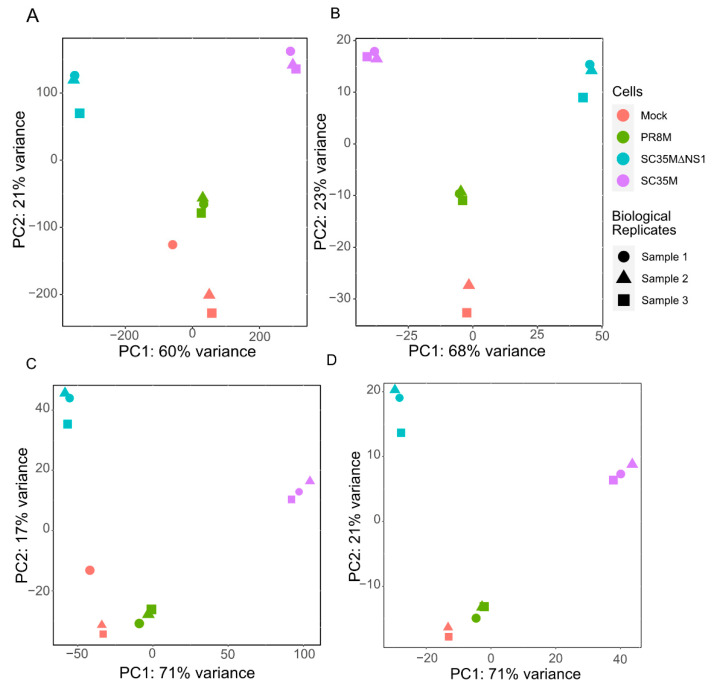
Principal component analysis (PCA) plots of the gene matrix (**A**,**B**) and HERV loci matrix (**C**,**D**). (**A**,**C**) PCA plots with all samples included. (**B**,**D**) PCA plots with the control sample 1 removed. Only one technical replicate is shown in all plots. See Appendix A (Figure A1 and Figure A2) for the gene matrices of the other two technical replicates.

After preprocessing and mapping the raw RNA-seq data, two count matrices were built (see Section 2.3 for the details). One matrix contains read counts of 26,423 genes and 81 HERV consensus sequences in different samples, while the other matrix contains read counts for 28,217 individual HERV loci. The principal component analysis (PCA) of the first matrix (Figure 1A,B) groups together the samples from the same cell line as well as the uninfected mock cells, except for one outlier sample of the uninfected control cell line, which was excluded (Figure 1B). PCA analysis of the second read count matrix yielded qualitatively similar results (Figure 1C,D), except that the PR8M infected cells and mock cells were close to each other, in favor of a closer expression pattern of HERVs in these two cells.

**Figure 2:**
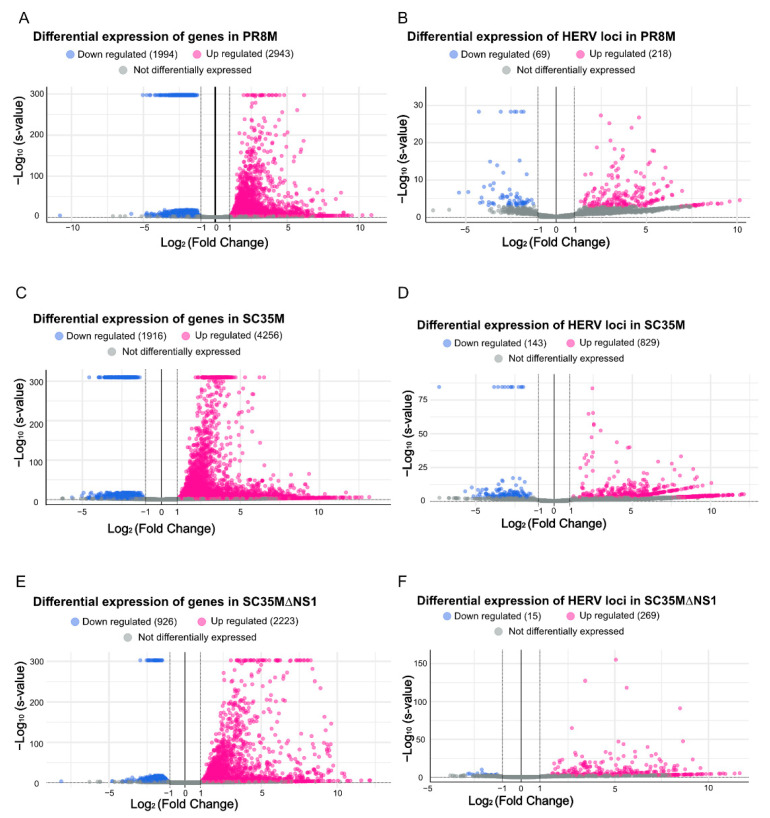
Volcano plots illustrating the differential expression of genes and HERV loci in all three cell lines compared to the mock control. (**A**,**B**) Differential expression genes and HERV loci in PR8M. (**C**,**D**) Differential expression of genes and HERV loci in SC35M. (**E**,**F**) Differential expression genes and HERV loci in SC35MΔNS1. DESeq2 converts very small s-values to 0, which leads to 580 genes in PR8M, 343 in genes in SC35M, and 75 genes in SC35MΔNS1 all having the same -Log10 (*s*-value) and appearing at the top.

**Figure 3:**
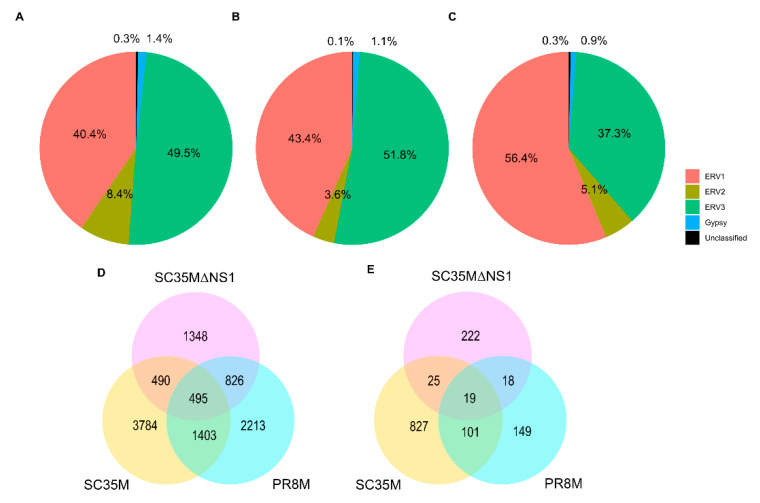
(**A**–**C**) Percentage of DEHERVs in each HERV superfamily in the cells infected by PR8M (**A**), SC35M (**B**), and SC35MΔNS1 (**C**). (**D**,**E**) Venn diagrams showing the overlap between the differentially expressed genes (**D**) and HERV loci (**E**) in the three infected cells (SC35M, SC35MΔNS1, and PR8M) relative to mock control.

**Figure A3:**
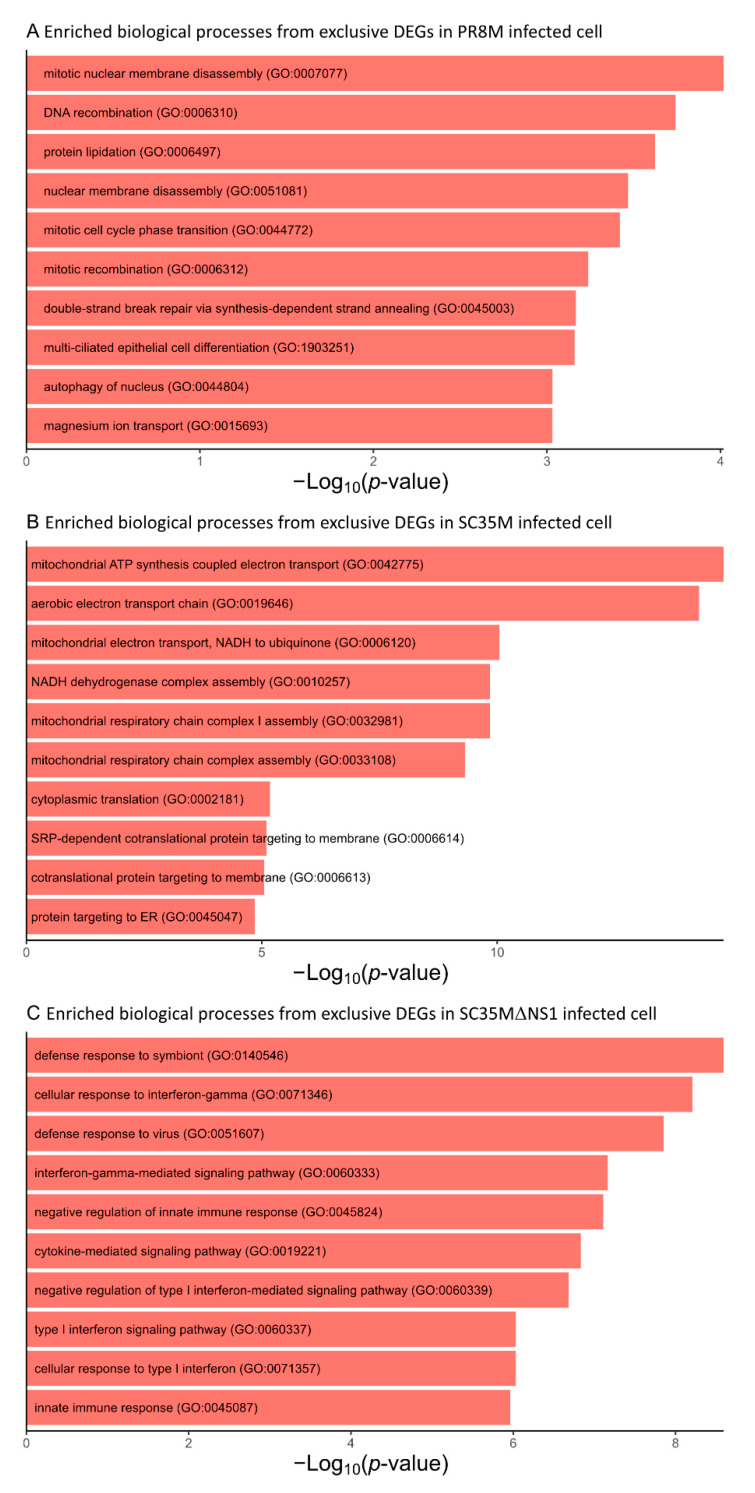
Top 10 most significantly enriched biological process GO terms associated with the genes exclusively differentially expressed in (**A**) PR8M infected cells, (**B**) SC35M infected cells, (**C**) SC35MΔNS1 infected cells. The terms are ranked according to the *p*-value.

The differential expression analysis of genes (DEGs) and HERVs (DEHERVs) between the three infected cell types and the mock control was conducted using the DESeq2 R package [33]. The volcano plot in Figure 2 gives the numbers of significantly (s-value < 1 × 10^−3^, Log2 fold change > ±1) differentially expressed genes (DEGs) and HERV loci (DEHERVs). Overall, the majority of DEGs (Figure 2A,C,E) and DEHERVs (Figure 2B,D,F) were up-regulated in the infected cells as compared to the non-infected control cells. The majority of the DEGs were constituted by protein-coding genes (86% in PR8M, 75% in SC35M and 90% in SC35MΔNS1), with the second-most abundant gene type being long non-coding RNAs (10% in PR8M, 15% in SC35M, and 6% in SC35MΔNS1) (Table S3). There was no dominant differentially expressed HERV type across all three infected conditions. In PR8M and SC35M, the biggest portion of DEHERVs belongs to the ERV3 group, while that in SC35MΔNS1 is ERV1 (Figure 3A–C). Most of the DEHERVs in PR8M and SC35M are mammalian apparent LTR retrotransposons (MaLRs), while in SC35MΔNS1, more HERV loci from the HERVW9 group were differentially expressed (Table S4). There are 495 common genes that were differentially expressed in all three infected cells compared to the mock cells (Figure 3D, Table S5). Interestingly, these common DEGs are enriched in biological processes or pathways associated with viral immunity (Table S5) according to the gene ontology (GO) and Kyoto Encyclopedia of Genes and Genomes (KEGG) enrichment analysis. When considering each cell pair, the overlap of either DEGs (Figure 3D) or DEHERVs (Figure 3E) was below 50%, indicating substantial differences between the cell lines infected by the different viruses. According to the GO enrichment analysis (Figure A3), the top 10 most significantly enriched biological process (BP) terms derived from the genes differentially expressed exclusively in SC35MΔNS1 are all linked to the immune system, while in the other two cell types, no immune-related biological processes appeared among the top 10 most significantly enriched GO terms.

### 3.2. Analysis of Specific DEHERV Types and Their Association with Genes

In addition to the locus-specific DE analysis of HERVs, we employed DESeq2 to analyze the overall expression tendency of the 81 HERV element types with non-zero expression levels. Table S6 lists differentially expressed HERV elements up-regulated upon IAV infection in each infected cell. LTR12C, along with its associated internal part HERV9NC-int, which belongs to the HERVW9 group, was up-regulated in all three infection conditions. Similarly, LTR5 and LTR5_Hs from the HML-2 class (but not their internal portion HERVK-int) were up-regulated in the IAV-infected cells. HERVH-int, along with its associated LTR7B, LTR13, and its variant LTR13A (associated LTRs of HERVK9-int) and LTR10C (associated LTR of HERVI-int) were only up-regulated in PR8M and SC35M but not in SC35MΔNS1. In summary, except for a few HERV types up-regulated in all three infected cells, infection with the two wild-type strains caused up-regulation of similar and more diverse sets of HERV groups than in the mutant strain.

We further explored the association between DEGs and the identified up-regulated HERV-LTRs, which can harbor regulatory elements such as enhancers and promoters [41,42,43] and may affect genes up to 100 kbp away both upstream and downstream [44]. Therefore, we constructed a series of Fisher’s tests to determine whether up-regulated DEGs show enrichment for certain specific types of up-regulated LTR loci within the 100kb vicinity of their TSS as compared to all genes. A positive association between DEGs and DEHERV loci was judged based on significant (adjusted *p*-value < 0.05 for test series) odds values of the Fisher’s test larger than 1. Out of the 18 HERV-LTRs types considered in this work, we identified five and one types of up-regulated LTRs positively associated with DEGs in SC35M and SC35MΔNS1, respectively (Table S7; see Table 2 for an example). LTR12C is the only positive result from SC35MΔNS1, which is also among the five positive results from SC35M. The analysis of the PR8M infected cells did not yield significant results. We further performed HOMER motif analysis on the up-regulated HERV loci for the 18 HERV-LTRs [37]. In all three infected cells, the most enriched motif in up-regulated HERV loci was the NFY(CCAAT) motif, known as a promoter. Other enriched motifs can be found in the Supplementary Materials MotifAnalysis.zip.

### 3.3. Pairs of Differentially Expressed HERVs and Genes (DEGERV-G)

**Figure 4:**
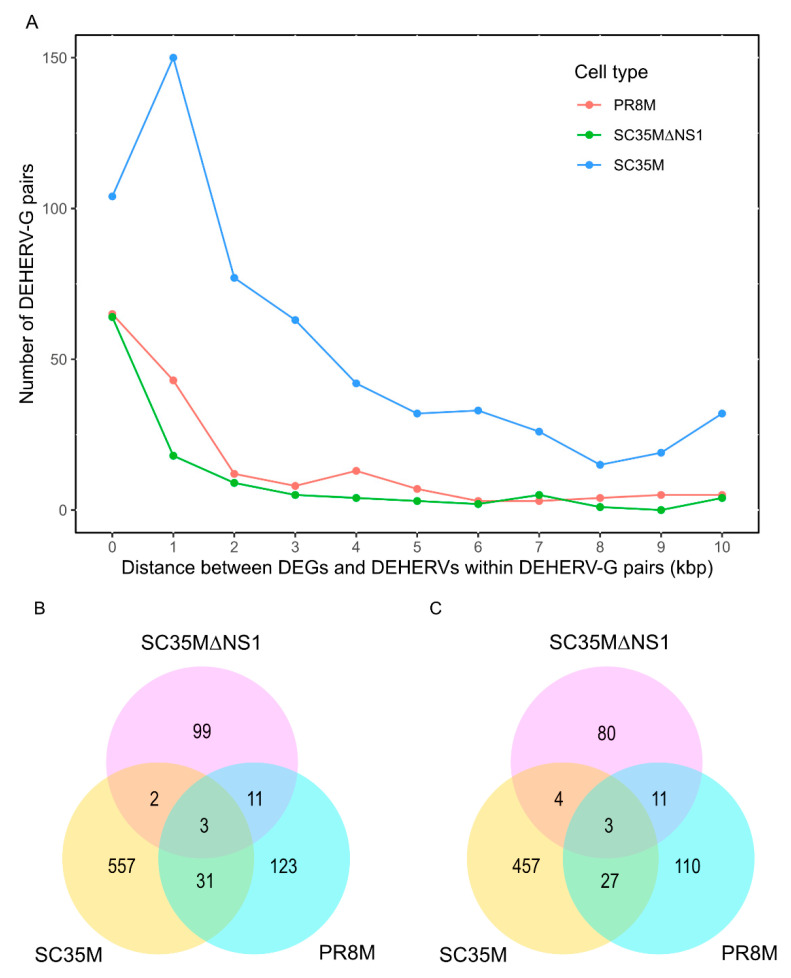
(**A**) Distribution of distances between DEGs and DEHERVs within DEHERV-G pairs. Zero distances correspond to overlapping DEGs and DEHERVs. (**B**,**C**) Overlaps between DEHERV-G pairs (**B**) and genes involved in DEHERV-G pairs (**C**) among the three IAV infected conditions vs. mock.

For each DE HERV locus, we identified the nearest DE gene within 100kbp on the same strand [22], and such HERV/gene pairs were regarded as DEHERV-G pairs. A total of 168, 593, and 115 DEHERV-G pairs were identified in PR8M, SC35M, and SC35MΔNS1 cells, respectively (Table S8). In line with the results reported by Wang et al., most pairs were constituted by up-regulated genes and HERVs (Table 3). All infected conditions showed a general tendency of decreasing DEHERV-G pair counts with the increasing relative distance between DEGs and DEHERVs (Figure 4A). Very few DEHERV-G pairs or genes involved in DEHERV-G pairs were common to all three cell types, with SC35MΔNS1 exhibiting the smallest overlap with other cells (Figure 4B,C, Table S8).

**Figure 5:**
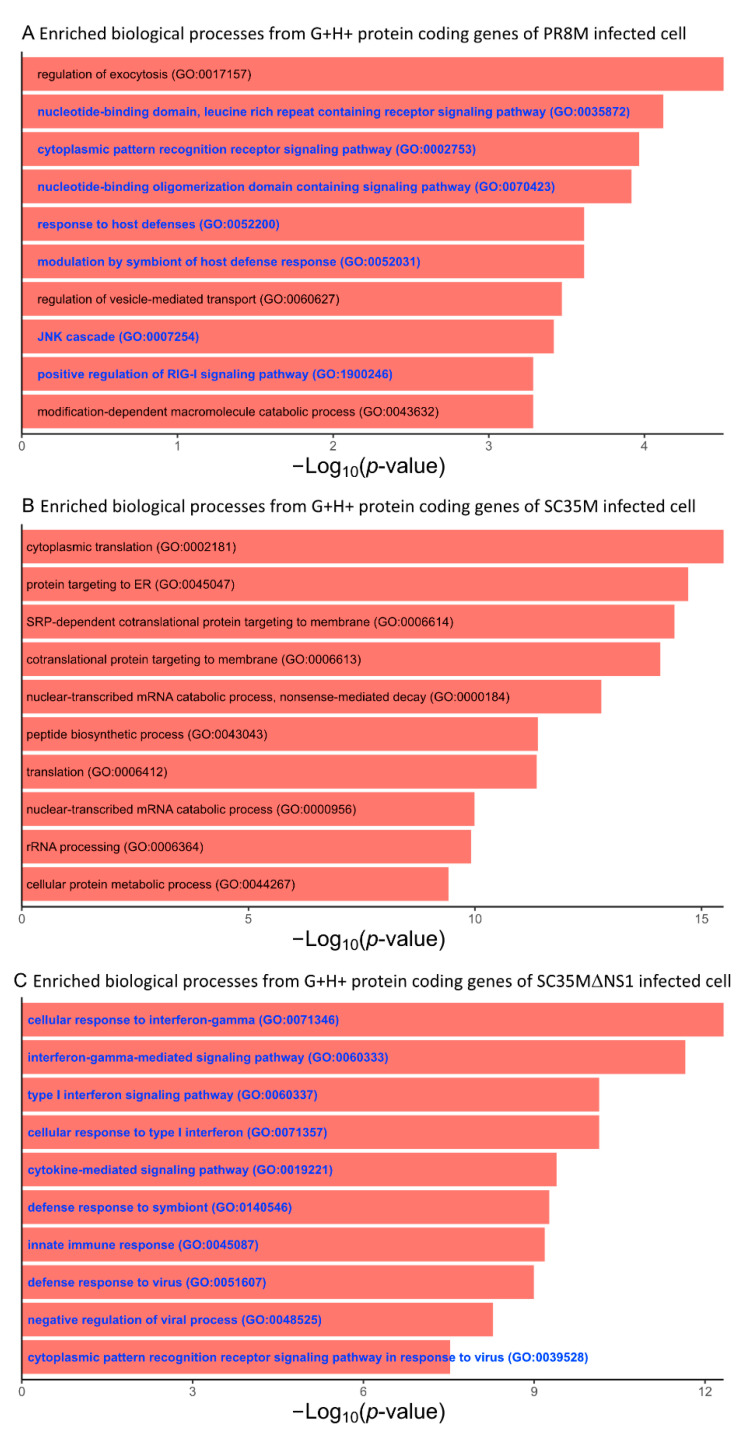
Top 10 most significantly enriched biological process GO terms associated with the protein-coding genes of the G+H+ DEHERV-G pairs. Terms are ranked according to the *p*-value. (**A**) PR8M infected cells, (**B**) SC35M infected cells, and (**C**) SC35MΔNS1 infected cells. Go terms related to immune or inflammation processes are shown in blue color.

**Figure 6:**
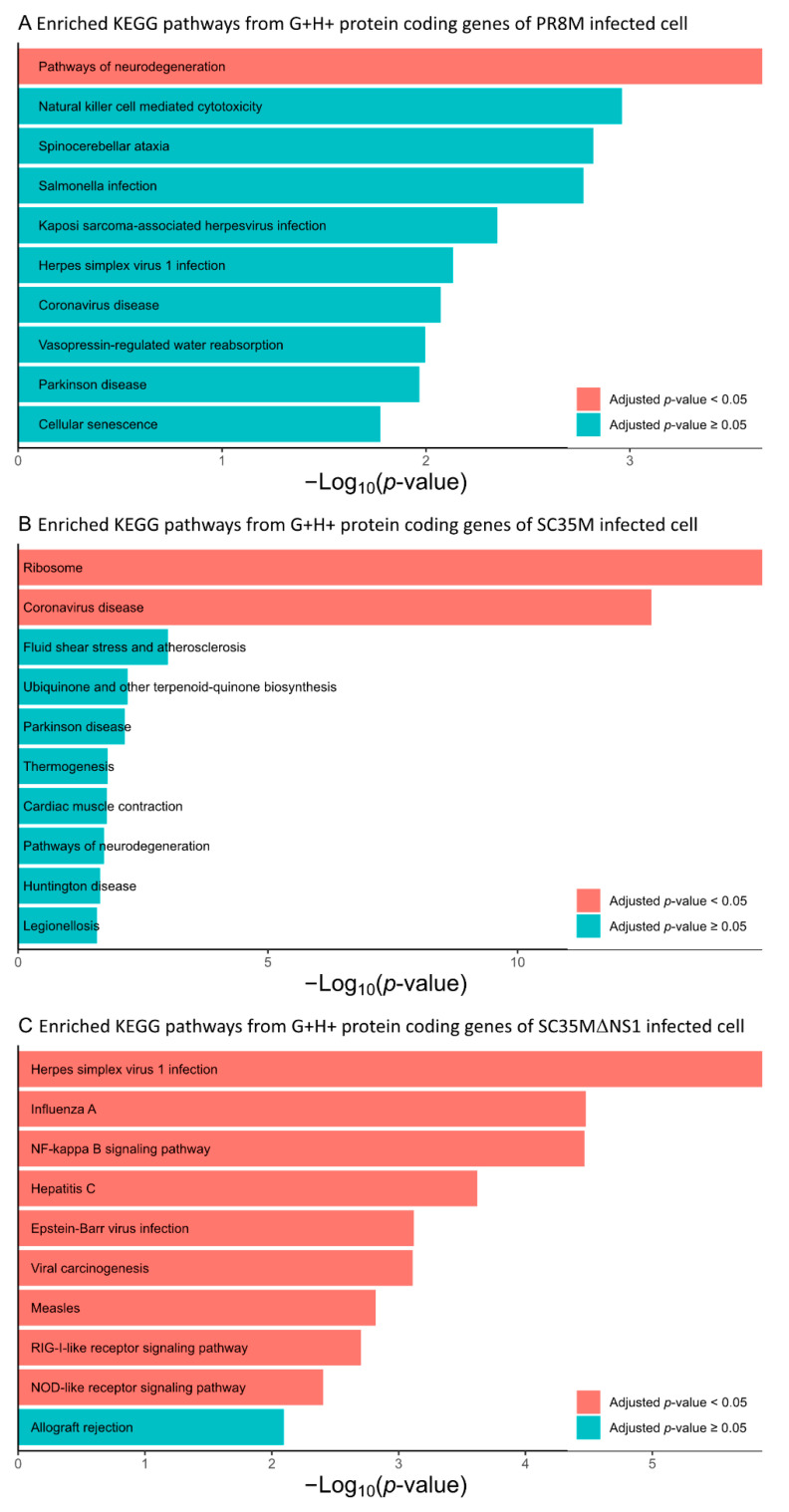
Top 10 most significantly enriched KEGG pathways associated with the protein-coding genes of the G+H+ DEHERV-G pairs. Terms are ranked according to the *p*-value and bars filled in blue means their *p*-value lower than 0.05 while Benjamini–Hochberg corrected *p*-values larger than 0.05. (**A**) PR8M infected cells, (**B**) SC35M infected cells, (**C**) SC35MΔNS1 infected cells.

We further performed a GO and KEGG enrichment analysis of the protein-coding genes involved in the G+H+ DEHERV-G pairs, i.e., those DEHERV-G pairs in which both genes and HERVs were up-regulated (Table S9). This analysis revealed that the top 10 most significantly enriched BP terms derived from the proteins involved in DEHERV-G pairs in the SC35MΔNS1 infected cells were all related to the cellular immune response or inflammation (Figure 5C). Similarly, more than half of the top 10 enriched GO terms are related to immune or inflammation processes in PR8M (Figure 5A). By contrast, none of the top 10 enriched GO terms in SC35M’s are directly linked to the immune or inflammation process (Figure 5B). The KEGG enrichment analysis yielded one, two, and nine significantly (Benjamini–Hochberg corrected *p*-values < 0.05) enriched pathways in PR8M, SC35M, SC35MΔNS1, respectively (Figure 6). The proteins from the SC35MΔNS1’s DEHERV-G pairs were enriched in virus-associated pathways (including “Influenza A pathway”), while in the other two cell types, only one virus-related pathway was significantly enriched (“Coronavirus disease” in SC35M). In addition, three signaling pathways significantly enriched in SC35MΔNS1’s DEHERV-G proteins -“NF-kappa B signaling pathway”, “RIG-I-like receptor signaling pathway”, and “NOD-like receptor signaling pathway”—are all related to immune activities and inflammation [45,46,47].

## 4. Discussion

Similar to most viral infections, IAV causes inflammation upon infection of the host [1]. The inflammatory response is an important step against IAV infection as it prevents virus replication and thus the spread of the virus [1]. However, an uncontrolled inflammatory response can cause lung damage and is often associated with severe cases of IAV infection [1]. Therefore, understanding the underlying mechanisms triggering antiviral inflammation upon IAV infection is of crucial importance. IAV is initially recognized by pattern recognition receptors (PRRs), including toll-like receptors (TLR), nod-like receptors (NLRs), and retinoic-acid-induced-gene-1-like receptors (RIG-1) [48]. It has also been reported that different HERV groups can influence inflammation via different mechanisms, including innate sensing via ERV-derived nucleic acids and PRRs [49,50,51,52,53]. In addition, HERV LTRs can also act as promoters or enhancers to control the expression of inflammatory genes [2,6,21]. Many recent studies have provided compelling evidence that HERVs critically influence gene networks [3,6,21,54], and it has been shown that several HERV groups can be reactivated upon infection with exogenous viruses, including HIV-1, hepatitis, and IAV [9,10,11,13]. The primary goal of this study was to assess the effect of IAV on HERV expression and its influence on host gene networks, potentially impacting the antiviral immune response. We investigated the reactivation of HERV expression in A549 infected IAV cells, focusing on pairs of differentially expressed HERVs and their nearest differentially expressed human genes (within 100 kb) on the same strand (DEHERV-G pairs [22]). We identified several HERV elements, including HERV LTRs, differentially expressed upon IAV infection, as well as genes associated with differentially expressed HERVs, which are associated with antiviral immunity. In particular, we found that HERV elements LTR5_Hs, LTR5, LTR12C, and HERV9NC-int are significantly up-regulated in PR8M, SC35M, and SC35MΔNS1 infected A549 cells. Interestingly, motif analysis revealed that the up-regulated loci of these HERV elements in all three infected cells are enriched in the NFY(CCAAT) motif, known to serve as a promoter, which implies that the transcription factor NFY might be responsible for the reactivation of identified HERV LTRs. Moreover, elements containing LTR5_Hs, LTR5, LTR5A, and LTR5B belong to the HERV-K(HML-2) subgroup [55,56]. Several HERV-K(HML-2) proviruses still encode the retroviral proteins gag, pol, and env [57]. HERV-derived proteins have already been demonstrated to impact immune activation via several mechanisms [2,3,5]. The HERV-K(HML-2) dUTPase has been described to trigger a TH1 and TH17 cytokine response as it activates NF-κB via TLR2 as well as several other cytokines in humans [58]. In addition, the HERV-K (HML2)-encoded protein Rec has a Rev-like function, as it stabilizes un-spliced or incompletely spliced viral transcripts and enhances their nuclear export [59,60]. In the early human embryo, Rec is proposed to induce an antiviral state by activating innate immune pathways and increasing the interferon-induced transmembrane protein 1 to inhibit viral infection [61]. HERV-K(HML-2)-derived gag proteins have also been described to co-assemble with HIV-1 gag and thus reduce HIV-1 infectivity as well as virion release [62,63]. Even though HIV-1 and influenza are very different, HERV and IAV domains might nevertheless interact with each other to affect IAV virus replication.

In addition to LTR5 elements, we also observed the activation of LTR12C elements upon IAV infection. LTR12C elements belong to the ERV9 group and are present in some 2500 copies in the human genome [2]. HIV-1 infection results in the upregulation of several LTR12C elements, which act as promoters for the interferon-inducible guanylate-binding proteins 2 (GBP2) and 5 (GBP5) [2]. GBP2 and GBP5 are well-known restriction factors with broad antiviral activity against viral replication, including Zika virus, HIV-1, measles virus, as well as Sars-CoV2 [64,65,66]. Thus, LTR12C might serve as an alternative promoter and impact the expression of GBP2 as well as GBP5 if reactivated upon IAV infection. Excitingly, GBP5 was also among the genes identified in DEHERV-G pairs (Table S8), suggesting the regulation of GBP5 by LTR12C upon IAV infection. However, further functional analyses are needed to prove this hypothesis. Interestingly, the second most abundant gene type identified in this study upon IAV infection among all viral strains belonged to lncRNAs. ERV-derived lncRNAs can impact antiviral immune response, e.g., by regulating key regulators of NF-κB signaling. Here, infection with RNA viruses, such as Sendai virus or vesicular stomatitis virus, resulted in the upregulation of an ERV-derived lncRNA, which facilitated the expression of the NF-κB subunit RELA [20]. RelA, also known as p65, is part of the NF-κB homo/heterodimers and possesses a transcription activation domain that is important for activating target gene expression and thus plays a critical role in antiviral immune response [67]. Further research is needed to understand the potential impact of lncRNAs on immunological processes.

HERV-derived nucleic acids can also trigger innate sensing pathways via PRRs. Just recently, it has been sown that the activation of IAV results in the loss of SUMO-modified TRIM28 [49], which is described as a key regulator of HERV expression [49,68,69,70]. The loss of SUMOylated TRIM28 resulted in the derepression of several ERV groups during IAV infection and induced an IFN-mediated innate immune response via RIG-I, MAVS, TBK1, as well as JAK1 signaling [49]. However, in the presence of the viral dsRNA-binding protein NS1, the induction of IFN-stimulated genes by ERV-derived dsRNAs was limited [49]. This suggests that NS1 might regulate ERV-derived dsRNAs and thus help to control the antiviral immune response pathways upon IAV infection. Furthermore, in this study, we identified differences in the activation of different HERV elements upon IAV infection in addition to infection with an NS1-lacking IAV strain. Overall, IAV infection, independent of the NS1 protein, resulted in the upregulation of four types of HERV elements—LTR5_Hs, LTR5, LTR12C, and HERV9NC-int—three of which have already been described to be involved in immunity as explained in the sections above. However, the activation of the HERV types, LTR13, LTR5A, LTR7B, LTR10C, as well as HERVH-int, was only observed upon infection with WT IAV strains but not with the delta NS1 strain. Some of these HERV groups, such as LTR5A and LTR7B, are known to have the potential to generate dsRNAs or lncRNAs [14,20,71,72] and, therefore, might be involved in the regulation of the antiviral immune response. In this context, it would be interesting to investigate in future experiments if NS1, which can oligomerize around long as well as short dsRNAs via a sequence-independent dsRNA-binding activity, might regulate these specific HERV groups to manipulate antiviral immune response upon IAV infection [73].

In summary, our study, among others [74], identified several HERV elements (DEHERVs) as well as genes (DEGs) differentially regulated upon IAV infection. Interestingly, several identified DEGs in close proximity to analyzed DEHERVs and both up-regulated after IAV infection (DEHERV-G pairs) were associated with immune responses such as defense responses to viruses and the regulation of innate immune responses. Previous studies have already shown that numerous HERV groups have the potential to act as interferon enhancers and promote the evolution of transcriptional networks under the interferon response [3]. Moreover, our findings imply that specific HERV groups/elements, up-regulated after IAV infection, might have the potential to trigger specific gene networks and influence host immunological pathways as well as antiviral immune responses. In the future, functional studies will be needed to decipher the exact mechanisms that lead to the activation of HERV immune networks upon viral infection, as well as help to uncover new strategies that viruses have evolved to circumvent HERV-mediated immunity.

## Figures and Tables

**Table 1:** Model of the second Fisher’s exact test for a given LTR type (using LTR13 as an example).

Model of second Fisher’s test	Specific DE HERV-LTR (e.g., LTR13) appearing within 100 kbp of DEG TSS more frequently than expected by chance	Specific DE HERV-LTR (e.g., LTR13) not appearing within 100 kbp of DEG TSS more frequently than expected by chance
Significantly differentially expressed genes	Number of DEGs having LTR13 within 100 kbp of their TSS	Number of DEGs having no LTR13 within 100 kbp of their TSS
Genes that were NOT significantly differentially expressed	Number of non-regulated genes having LTR13 within 100 kbp of their TSS	Number of non-regulated genes having no LTR13 within 100 kbp of their TSS

**Table 2:** Data on LTR12C occurrence used in the Fisher’s test. The total number of genes considered in this test is 26423. See Methods for details on Fisher’s test model.

	PR8M	SC35M	SC35MΔNS1
Number of up-regulated DEGs having up-regulated LTR12C loci within 100 kbp of their TSS	10	26	22
Number of genes having up-regulated LTR12C loci within 100 kbp of their TSS	59	84	122
Number of up-regulated DEGs	2943	4256	2233
Fisher’s test odds value	1.63	2.34	3.25
Fisher’s test *p*-value	0.15	8.34 × 10^−4^	8.74× 10^−7^
Adjusted *p*-value	1	3.54× 10^−3^	1.49× 10^−5^

**Table 3:** Differential expression status of DEHERV-G pairs in the three cell types. Symbols +/− denote up/down-regulation of genes or HERVs, respectively. e.g., G+H+ indicates pairs in which both a HERV locus and a gene are up-regulated.

Cells	DEHERV-G Pairs				Involved Genes	
	G+H+	G+H−	G−H+	G−H−	Up Regulated	Down Regulated
PR8M	103	8	26	31	97	54
SC35M	451	32	84	26	391	100
SC35MΔNS1	104	1	5	5	88	10

**Figure A1:**
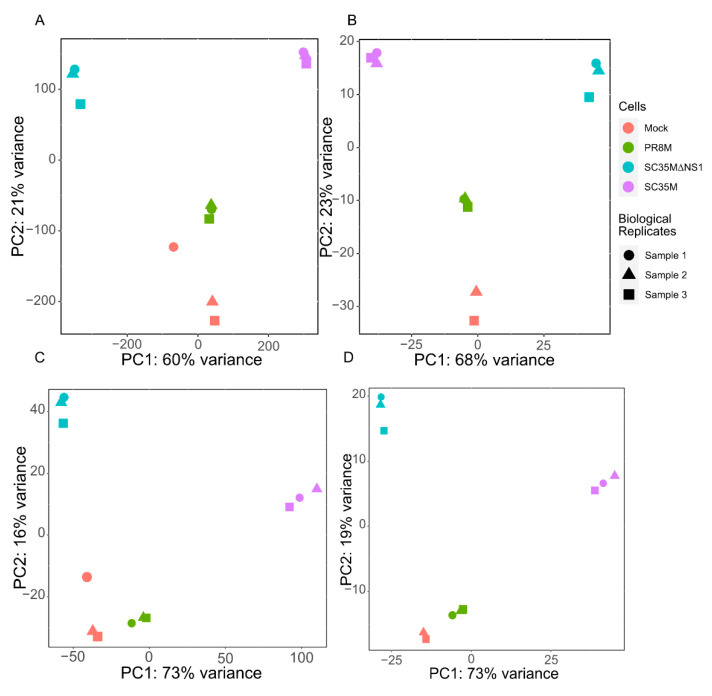
Principal component analysis (PCA) plots of the gene matrix (**A**,**B**) and HERV loci matrix (**C**,**D**) of second technical replicates. (**A**,**C**) PCA plots with all samples included. (**B**,**D**) PCA plots with the control sample 1 removed.

**Figure A2:**
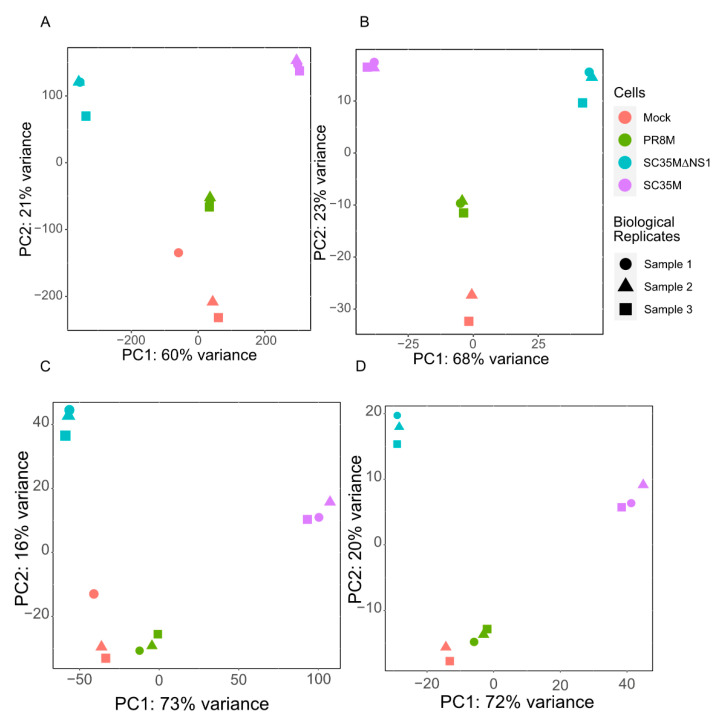
Principal component analysis (PCA) plots of the gene matrix (**A**,**B**) and HERV loci matrix (**C**,**D**) of third technical replicates. (**A**,**C**) PCA plots with all samples included. (**B**,**D**) PCA plots with the control sample 1 removed.

## Data Availability

RNA sequencing data can be obtained from the European Nucleotide Archive (ENA) through accession number PRJEB54734.

## Supplementary Materials

The following supporting information can be downloaded at: https://www.mdpi.com/article/10.3390/v14071591/s1, Table S1: Association of HERV internal portions and LTRs; Table S2: HERV LTR types involved in the analysis of the association between up-regulated HERV-LTR types and DEGs; Table S3: Counts of differentially expressed genes of each type; Table S4: Counts of differentially expressed HERV loci of each class; Table S5: Common differentially expressed genes in all three infected cells and enrichment analysis of these genes; Table S6: Differentially expressed HERV elements up-regulated upon IAV infection in each infected cell; Table S7: List of up-regulated HERV-LTR types that tend to appear within 100 kbp vicinity of up-regulated DEGs’ TSSs; Table S8: Detailed information about DEHERV-G pairs; Table S9: Protein coding genes involved in the G+H+ DEHERV-G pairs; MotifAnalysis.zip: All results from the HOMER motif analysis.
